# Retrospective analysis of the impact of anthracycline dose reduction and chemotherapy delays on the outcomes of early breast cancer molecular subtypes

**DOI:** 10.1186/s12885-018-4365-y

**Published:** 2018-04-20

**Authors:** Sigita Liutkauskiene, Saulius Grizas, Kristina Jureniene, Jorune Suipyte, Akvile Statnickaite, Elona Juozaityte

**Affiliations:** 1 Oncology Institute of Lithuanian University of Health Sciences, Kaunas, Lithuania; 2 Clinic of Surgery of Hospital of Lithuanian University of Health Sciences, Kaunas, Lithuania; 3 Cardiology Institute of Lithuanian University of Health Sciences, Kaunas, Lithuania; 4 Medical Academy, Faculty of Medicine, Lithuanian University of Health Sciences, Kaunas, Lithuania

**Keywords:** Breast cancer, Chemotherapy scheme modifications, Molecular subtypes, Overall survival

## Abstract

**Background:**

The objective of study was to determine the effect of anthracycline dose reduction and chemotherapy delays on 5-year overall survival in patients with stage I-III breast cancer, to establish the impact of molecular subtypes on the anthracycline modification effects and to analyze reasons for such chemotherapy scheme modifications.

**Methods:**

Medical records of patients with stage I-III breast cancer were reviewed. Inclusion criteria involved stage I- III breast carcinoma; radical surgery performed and 4 courses of AC regimen (doxorubicin and cyclophosphamide), or at least 6 courses of FAC regimen (fluorouracil, doxorubicin and cyclophosphamide) completed; no neoadjuvant chemotherapy applied; no taxane group medications administered; medical records maintain comprehensive data on treatment and follow-up. 5- year overall survival were analyzed using Kaplan-Meier and Cox proportional hazards models.

**Results:**

Significant 3.17 times higher death risk at 5 year period in patients who experienced anthracycline dose reduction compared with patients who did not experience any modifications was established (HR = 3.17, 95% CI 1.7–5.9, *p* < 0.001). Increased death risk in patients who experienced both chemotherapy dose reduction and treatment delays compared with patients who did not experience any modifications was also established (HR = 2.76, 95% CI 1.3–5.6, *p* < 0.05). 5- year overall survival was affected by anthracycline dose reduction by more than 15% in ER-HER2- group (80% *v*. 55.6%, *p* = 0.015), ER + HER2- group (90.7% *v*. 64.9%, *p* < 0.01) and ER+/-HER2+ group (100% *v.* 84.4%, *p* = 0.019). 5-year overall survival was affected by chemotherapy delays more than 2 cycles in ER-HER2- group (79.2% *v*. 51.4%, *p* = 0.002), ER + HER2- group (86.3% *v*. 58.8%, *p* = 0.014) and there was no difference in ER+/-HER2+ group. Main reasons for chemotherapy scheme modifications (in decreasing order) were the following: neutropenia, modifications with no objective medical reasons, thrombocytopenia, anaemia, fatigue, infection.

**Conclusions:**

Anthracycline dose reduction in patients with stage I- III breast cancer were associated with higher mortality risk and significantly decreased 5- year absolute survival in all molecular subtypes. Chemotherapy delays alone were not associated with decreased survival only in HER2 positive subtype. The most common reason for dose reduction or chemotherapy delays was neutropenia.

**Electronic supplementary material:**

The online version of this article (10.1186/s12885-018-4365-y) contains supplementary material, which is available to authorized users.

## Background

Meta-analyses of multiple studies established that adjuvant chemotherapy improves survival of patients with early stage breast cancer. In all meta-analyses involving taxane-based or anthracycline-based regimens, proportional risk reductions were affected by age, nodal status, tumour diameter, differentiation grade, oestrogen receptor status, or tamoxifen use [1]. However, it is important to note that these meta-analyses included randomised clinical trials, where chemotherapy modification is impossible or extremely rare. Although in daily clinical practice, it is very common to adjust chemotherapy dose to avoid treatment-related complications. Retrospective study involving 1243 centres in USA of prevalence and reasons for dose adjustment of early stage breast cancer adjuvant chemotherapy showed that dose reductions ≥15% occurred in 36.5% of patients, and there were treatment delays ≥7 days in 24.9% of patients, resulting in 55.5% of patients receiving RDI less than 85% [2]. Clinical studies show that dose reduction and chemotherapy delay in treatment of chemotherapy sensitive tumours – non-Hodgkin lymphoma [3, 4], early stage breast cancer [5], advanced ovarian cancer [6] – are related to lower survival rates. Later clinical studies proved negative prognostic effect of reduced planned dose of chemotherapy for early stage breast cancer on patients’ survival [7, 8]. This raises a question in daily practice: is it worth to administer adjusted chemotherapy regimen, knowing its negative prognostic effect on survival?

The aim of our study was to determine the effect of anthracycline dose reduction and chemotherapy course delay on 5-year overall survival of women with early stage breast cancer, to evaluate the effect of chemotherapy modification on breast cancer molecular subtypes.

## Methods

This study was approved by the Bioethics Center of Lithuanian University of Health Sciences. We analysed medical documentations of patients with stage I-IIIA breast cancer, who underwent treatment in Affiliate of Hospital Lithuanian University of Health Sciences Kaunas Oncology Hospital in 2004–2007.

**Fig. 1:**
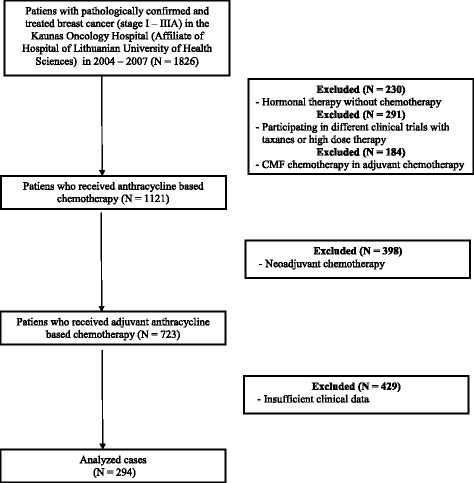
Flow chart of cases used for analysis. The whole process of identification of the patients that meet study criteria is shown in this chart. 294 patients were used for final analysis

Inclusion criteria of the study were the following: 1) patients with stage I-IIIA breast cancer diagnosed in 2004–2007; 2) radical tumour surgery performed; 3) adjuvant chemotherapy administered: either at least 4 courses of AC regimen (doxorubicin and cyclophosphamide), or at least 6 courses of FAC (fluorouracil, doxorubicin and cyclophosphamide) regimen; 4) no taxane group medications administered; 5) medical records maintain comprehensive data on treatment and follow-up. Exclusion criteria: 1) neoadjuvant chemotherapy; 2) treatment with taxane group medications or high dose chemotherapy; 3) treatment with CMF (cyclophosphamide, methotrexate, fluorouracil) chemotherapy regimen; 4) non-complete medical documentation; 5) less than 4 courses of AC chemotherapy regimen or less than 6 courses of FAC chemotherapy regimen administered; 6) history of another neoplastic process. The process of cases selection is shown in Fig. 1.

We collected the following data: patient’s age at the moment of diagnosis, body surface area, menopausal status, tumour histology and differentiation, tumour stage at the moment of diagnosis, hormone receptor status, HER2 status, surgery date, chemotherapy regimen, administered dose of anthracycline, disease progression date, last follow-up date and date of death. Three variables were chosen to assess the anthracycline impact on 5 year survival: anthracycline dose reduction, when cumulative dose was reduced by more than 15% (> 15% v. ≤ 15%); the number of delayed days, when cumulative period of delay was more than 7 days (> 7 days v. ≤ 7 days) from the first cycle; the number of delayed cycles from the first cycle when the patient had more than 2 cycles with more 3 days of cycle delay with respect to the planned schedule (˃2 delayed cycles v. ≤ 2 delayed cycles).

Patients according hormone and HER 2 receptor status were divided into three molecular subtypes groups: hormone positive (Luminal A and B, ER + HER2-), HER2 positive (ER+/-HER2+) and receptor negative (ER-HER2-). Ki67 was not tested at that time.

We determined reasons for anthracycline dose modifications. It is important to note that none of the patients received granulocyte colony stimulating factors during adjuvant treatment.

### Statistical analysis

The main goal of the study was to assess the impact of the anthracycline dose modifications on 5-year overall survival (OS). OS at 5 years was defined as being alive 5 years after cancer diagnosis (the date of breast cancer surgery), with those alive censored at the last known follow up.

The Kaplan-Meier method was used for single variable survival data analysis. Differences of the survival rate were determined using the log-rank test.

The prognostic value of anthracycline dose modification on 5-year OS was evaluated using Cox’s proportional hazards regression model with adjustment for tumour stage, molecular subtype, and menopausal status (variables that are known to affect survival according Kaplan-Meier method). The proportional hazards assumption was examined by log(−log) plot of survival. The results are presented as hazard ratio (HR) for death risk during 5 year period after breast cancer diagnosis and 95% confidence intervals.

The SPSS software version 22 was used for statistical analysis.

## Results

Of the 1826 breast cancer patients included in the initial database, 294 were included in this retrospective analysis. 5-year overall survival of all included patients was 79.3%.

The impact of treatment period, tumour stage, lymph node involvement, menopausal status, hormone and HER2 receptor status, anthracycline chemotherapy modification on 5-year survival was tested conducting Kaplan Meyer analysis. We found statistically significant impact of tumour stage, molecular subtype, menopausal status and anthracycline treatment modification on 5-year overall survival (Table 1).

**Table 1:** Patients groups characteristics (*N* = 294)

Characteristics	n	5 year survival (%)	p overall (log rank test)
Treatment period			
2004	152	80.3	*p* = .42
2005	97	79.4	
2006	21	90.0	
2007	19	68.4	
Tumor stage			
I	67	83.2	*P* < .001 *pairwise comparisons* 1–3, **, 2–3 **
II	201	81.0	
III	26	50.0	
Lymph node involvement			
Not involved	179	82.0	*p* = .51
Involved	115	76.8	
ER and HER2 receptor status			
ER-HER2-	85	66.5	*p* = .002 *pairwise comparisons* 1–3 *, 3–4 * 1–4 **
ER-HER2+	17	87.5	
ER + HER2-	143	79.8	
ER + HER2+	49	93.9	
Menopausal status			
Pre/perimenopausal	94	85.3	*p* < .05
Postmenopausal	200	75.5	
Delayed number of cycles			
≤ 2 delayed cycles	187	86.3	*p < .001*
> 2 delayed cycles	107	65.4	
Delayed number of days at any cycle			
≤ 7 days	217	81.1	*p* = .093
˃7 days	77	71.8	
Dose reduction			
≤ 15%	225	89.7	*p* < .001
˃ 15%	69	65.4	
Anthracycline delay/reduction groups			
No anthracyclines reduction or delays	179	86.4	*p* < .001 *pairwise comparisons* 1–2, 1–4 ** 2–3, 3–4 * *1–3, 2–4 ns*
Anthracycline reduction only	38	57.6	
Anthracycline delays only	46	79.5	
Both anthracycline reduction and delays	31	60.2	

pairwise comparisons *p < .05, ** p < .001, ns – not signifficant

Patients were divided into four anthracycline delay / reduction groups. Chemotherapy delay group included patients with both chemotherapy delays more than 7 days and more than 2 cycles. There was no anthracycline dose reduction or chemotherapy delays in 179 patients (60.8%). In 115 patients (39.2%) chemotherapy scheme was modified: 38 patients (12.9%) experienced only anthracycline dose reduction, 46 patients (15.6%) experienced only chemotherapy delays and 31 patients (10.5%) experienced both anthracycline dose reduction and delays.

**Fig. 2:**
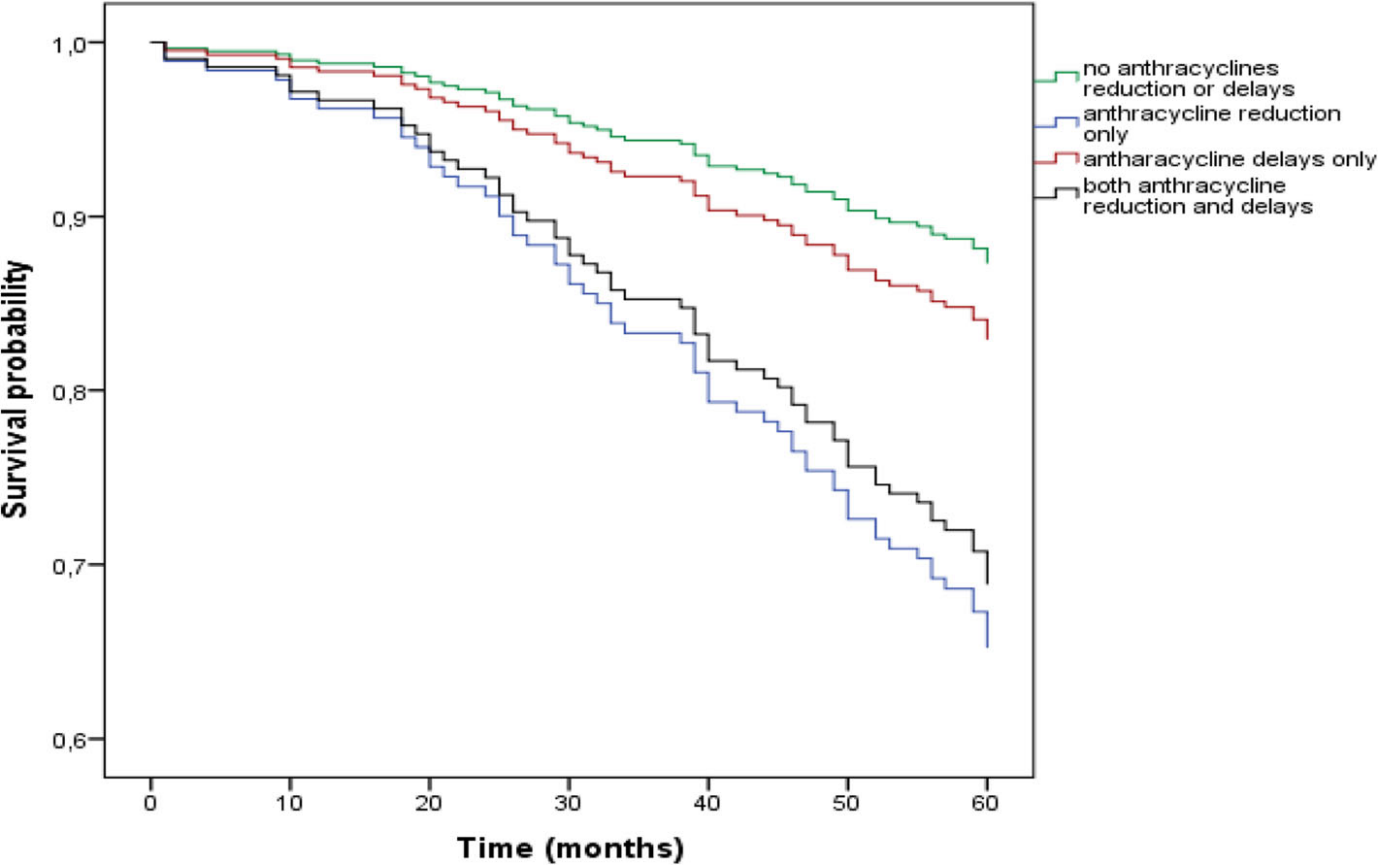
5-year survival: adjusted for tumor size, molecular subtype and menopausal status***.*** Cox proportional hazards model with adjustment to the tumor size, molecular subtype and menopausal status showed that mortality risk was 3.17 times higher in patients, who experienced only anthracycline dose reduction compared with patients who did not experience delays or dose reduction (HR = 3.17, 95% CI 1.7–6.0, *p* < 0.001), and also showed that mortality risk was 2.7 times higher in patients, who experienced chemotherapy delays and dose reduction compared with patients who did not experience delays and dose reduction (HR = 2.76, 95% CI 1.3–5.7, *p* < 0.05)

Cox proportional hazards model for 5- year overall survival with adjustment for tumor stage, molecular subtype and menopausal status revealed statistically significant difference in 5-year overall survival in patients who experienced anthracycline modification compared with patients who did not experience neither chemotherapy delays nor anthracycline dose reduction, e.g., death risk was 3.17 times higher in patients who experienced anthracycline dose reduction by more than 15% compared with patients who did not experience any modifications (HR = 3.17, 95% CI 1.7–6.0, *p* < 0.001). Significantly increased death risk in patients who experienced both chemotherapy dose reduction and treatment delays compared with patients who did not experience any modifications was also established (HR = 2.76, 95% CI 1.3–5.7, *p* < 0.05). Meanwhile chemotherapy delays did not have any statistically significant impact on 5-year overall survival, comparing to the patients who did not have any chemotherapy modifications. All data are presented in Fig. 2 and Additional file 1: Table S1.

**Fig. 3:**
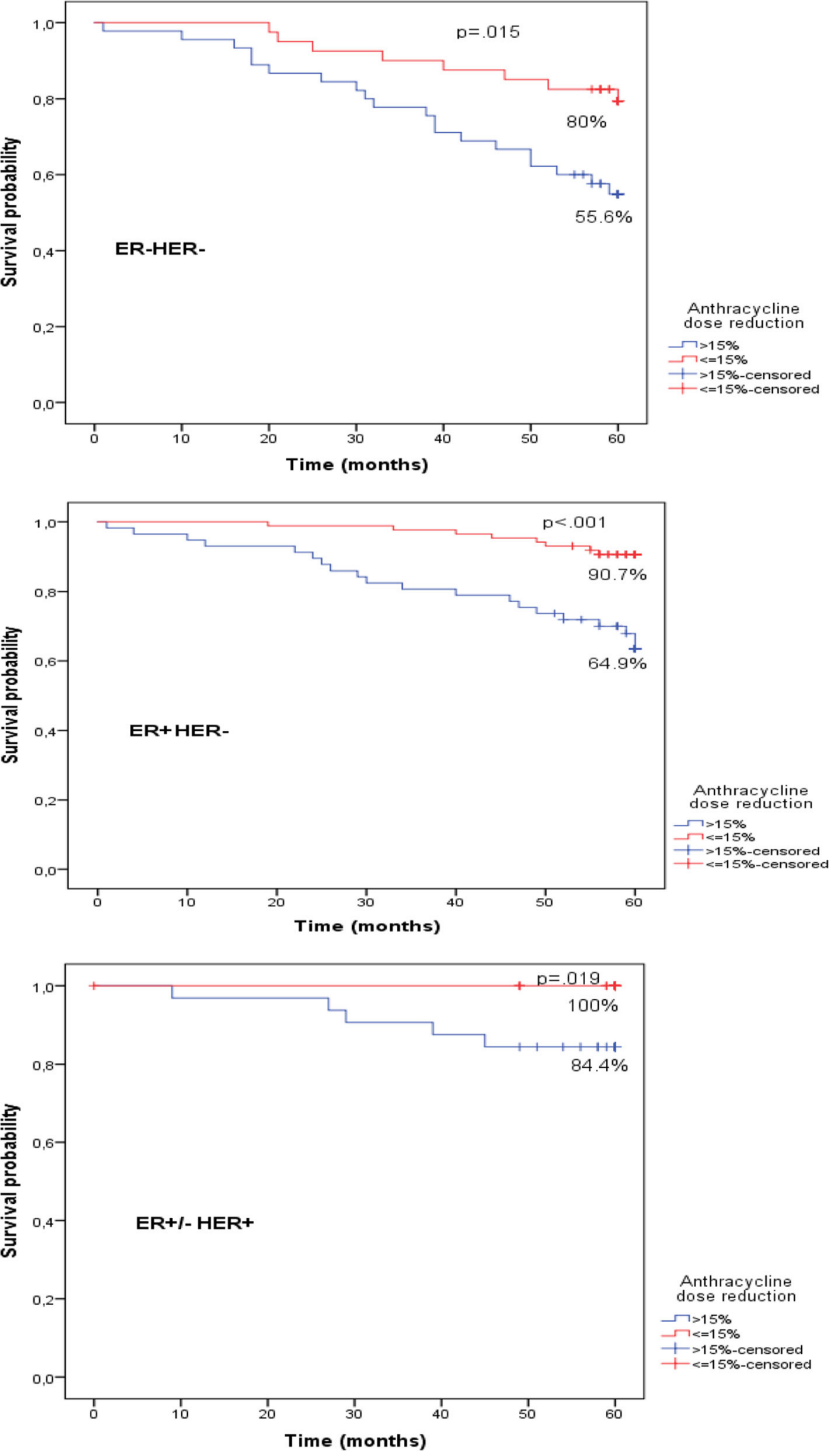
Impact of anthracycline dose reduction on 5-year survival in different molecular subtypes

**Fig. 4:**
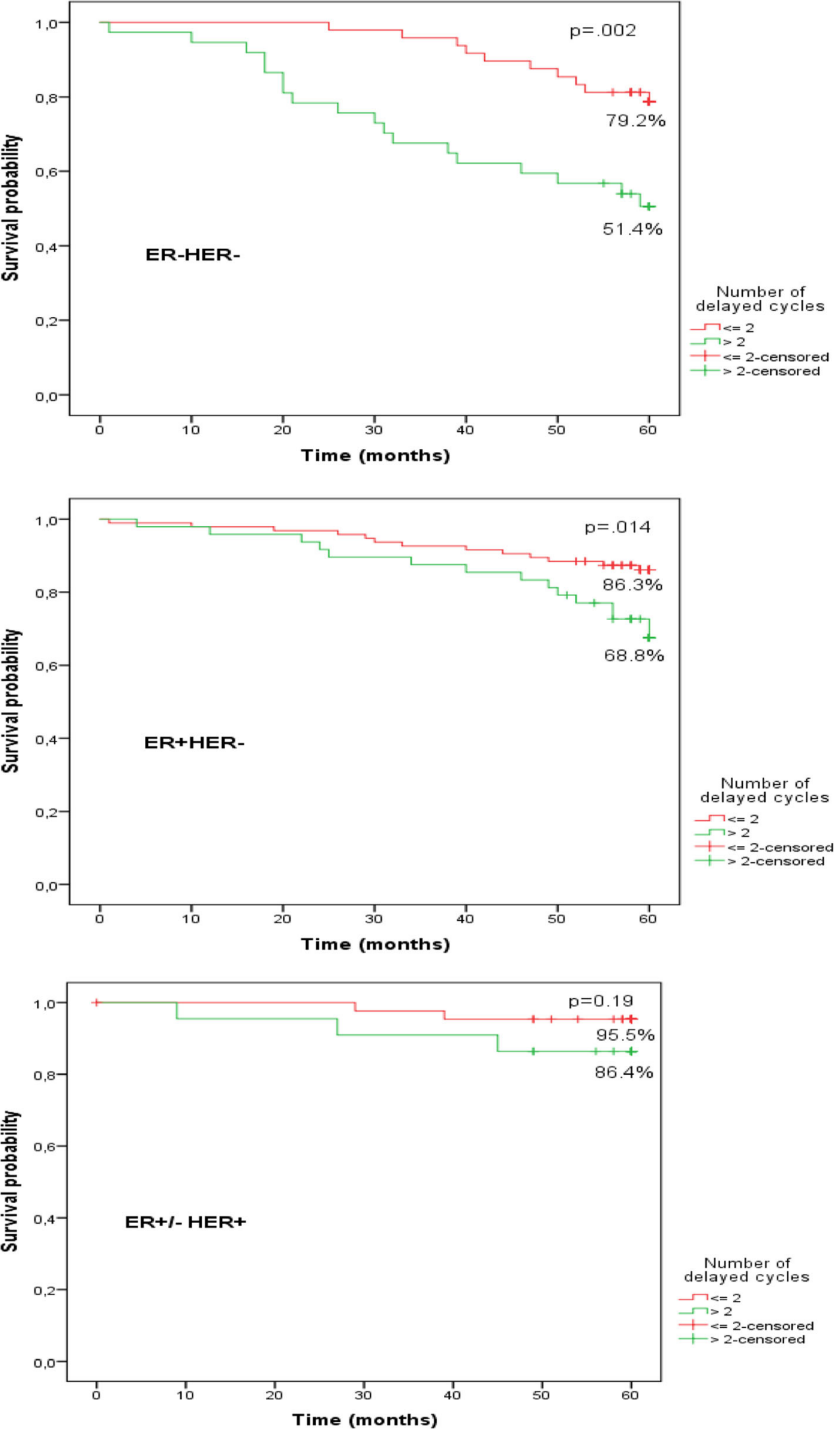
Impact of anthracycline delay on 5-year survival in different molecular subtypes

As chemotherapy course delay for more than 7 days had no statistically significant impact on overall survival (Table 1), we aimed to determine effect of anthracycline dose reduction and the number of delays on different molecular subtypes of breast cancer. All patients were categorized into three subgroups: ER + HER2-, ER-HER2- and ER+/-HER2+. 143 patients (49%) were diagnosed with ER + HER2- tumour: 48 patients (34%) experienced chemotherapy delays more than 2 cycles and 86 patients (60%) - anthracycline dose reduction more than 15%. 85 patients (29%) were diagnosed with ER-HER2- tumour: 37 patients (43%) experienced chemotherapy delays, and 40 patients (47%) - anthracycline dose reduction. 66 patients (22%) were diagnosed with ER+/-HER2+ tumour: 22 patients (33%) experienced chemotherapy delays, 34 patients (51%) - anthracycline dose reduction. We used Kaplan-Meier method to analyse effect of anthracycline dose reduction and chemotherapy delays on 5-year overall survival in different breast cancer subtype groups. We observed negative impact of anthracycline dose reduction on 5-year overall survival in all three groups (Fig. 3), and negative impact of more than 2 chemotherapy cycle delays on 5-year overall survival in ER + HER2- and ER-HER2- groups (Fig. 4).

**Fig. 5:**
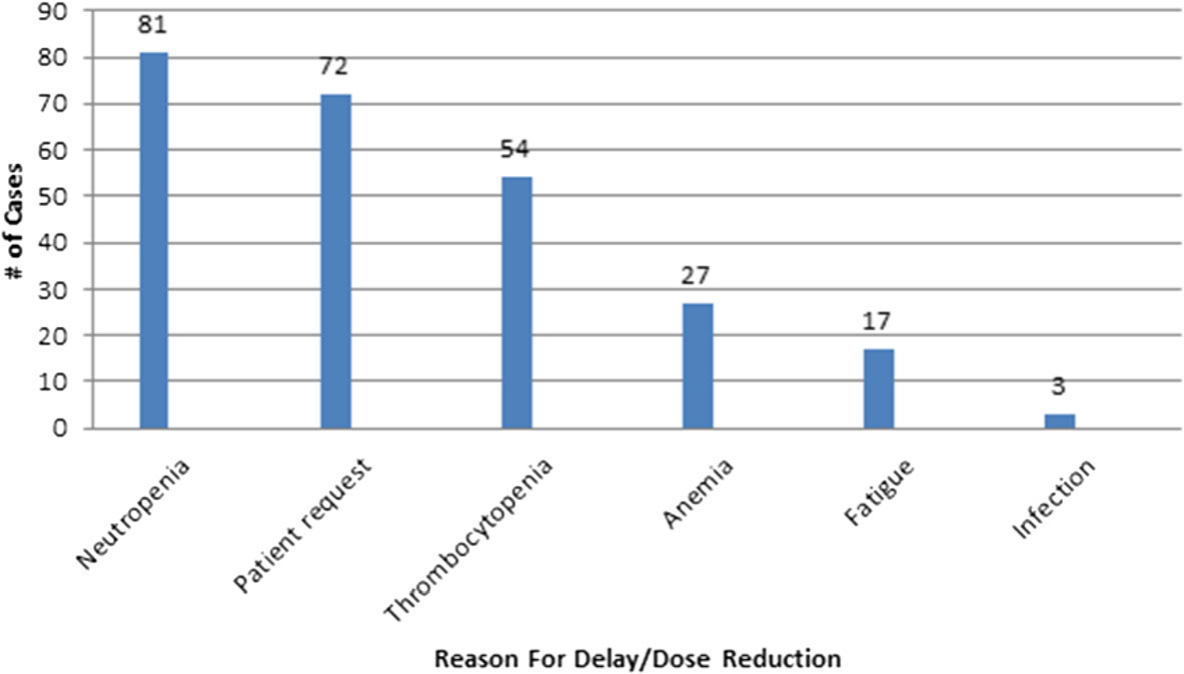
Reasons for chemotherapy delays and dose reductions

This study showed that usually there was more than one reason to modify chemotherapy, but the main medical reasons for chemotherapy scheme modifications were the following blood disorders: neutropenia (31.5%), thrombocytopenia (21.3%), anaemia (10.6%). Modifications with no objective medical reason accounted for 28.0% and was the second most frequent among all reasons of chemotherapy scheme modifications (Fig. 5). When medical records contained no medical reason that could predetermine anthracycline dose reduction or delay, such modification was considered as patient request and have no objective medical reason.

## Discussion

Meta-analysis published in 2012 shows that six months of anthracycline-based chemotherapy reduced the annual breast cancer mortality rate by approximately 21%, (RR 0.79 95% CI 0.72–0.85) and absolute 10 year gain was 6.5%**.** [1]. It is important that this meta-analysis evaluated results of various randomized clinical studies, where chemotherapy dose adjustment was not allowed or rare due to strictly inclusion criteria. Meanwhile, the aim of this retrospective study was to analyse prevalence of adjuvant systemic anthracycline chemotherapy modification and its impact on overall survival in real life. Retrospective studies show that in daily practice chemotherapy doses are adjusted for about 50% of all patients [2], in our study – 39.2%. Sometimes there was just one reason for chemotherapy modification, but more often there were multiple reasons, and the most common of them – neutropenia, what matches other retrospective clinical studies [6, 9]. Our data showed high percentage of not clinically grounded chemotherapy scheme modifications (28.0%). This was specified as modification with no objective medical reason as patients request, and this notion involves various social circumstances, transportation issues interfering patient’s admission at scheduled date, and unmotivated doctor’s decision to reduce recommended dose. This finding was very similar to the results of our earlier study [6]. However, Chan JK et al. presented the factors associated with the suboptimal treatment of women less than 55 years of age with early-stage ovarian cancer: women were more likely to live in poor neighborhoods, less likely to be seen by a gynecologic oncologist, and had more grade 1 and 2 tumors [10]. Non medical reasons can be avoided first of all by explaining the patients about negative impact of chemotherapy modifications on their clinical outcomes.

There are no general consensus of a precise definition about the length of treatment delays. We analysed of 7 and more days delays duration at any cycle and we didn’t find statistically significant impact on patient’s outcomes in our study. That duration maybe was too short to have negative impact on patient’s outcomes (Table 1). We used 10 days treatment delays duration in our previous retrospective analysis of the impact of platinum dose reduction and chemotherapy delays on the outcomes of stage III ovarian cancer patients [6], however Chirivella et al. assessed 15 days anthracycline delays duration in their study [8]. Negative impact on patient’s outcomes was shown in both aforementioned studies.

One of the main purposes of this study was to evaluate impact of chemotherapy modification on different subtypes of breast cancer. It came as a surprise, that delay of two and more chemotherapy courses insignificantly affected 5-year overall survival of women with HER2 positive tumour only, on the other hand it significantly affect survival of patients with ER-HER2- and ER + HER2- cancer subtypes.

Analysis of reduced chemotherapy dose on 5-year overall survival in different molecular subtypes showed the lowest, though still statistically significant negative impact in ER+/-HER2+ group. No doubt, this result is influenced by targeted therapy with Trastuzumab against HER2 protein and probably lower impact of chemotherapy itself in this subgroup. Results of our study support the statement of Prat A and colleagues - the intrinsic subtypes can help identify those patients with HER2+ early breast cancer that might be successfully treated without chemotherapy but with dual HER2 blockade since their tumours are fully sensitive to anti-HER2 therapy. Certainly, there is clinical evidence suggesting that these patients exist [11].

Cox analysis of impact of different chemotherapy modifications on overall survival was not surprising at all. It showed that dose reduction and combination of dose reduction and chemotherapy delay statistically significantly increased 5-year mortality rate. It seemed that chemotherapy delay alone did not affect mortality rate. However, Kaplan-Meier analysis of different molecular subtypes showed that chemotherapy delay is insignificant only in HER2 positive subtype (for women with ER+/-HER2+ type breast cancer), while it statistically significantly reduced survival for women with hormone and HER2 receptor negative tumor (ER-HER2-) and hormone receptor positive (ER + HER2-) tumours. This finding established the importance of group homogeneity and showed that interpretation of results might be different, if attention is not paid for this factor.

The major limitation of our study was the absence of information about Ki 67 level. This fact did not allow to analyse Luminal A and Luminal B molecular subtypes separately and determine impact of anthracyclines modifications on clinical outcomes of these molecular subtypes.

According to the data of our retrospective study it seems that it is not worth to administer adjusted chemotherapy regimen at all, because results showed its detrimental negative prognostic effect on survival: anthracycline dose reduction leads to decreased absolute 5 year survival by 16% in HER2 positive molecular subtype and 35% in both: hormone positive and hormone and HER2 negative subtypes. However, earlier meta-analyses showed only 6.5% 10 -year overall survival gain in patients treated with optimal chemotherapy. Dose reduction and chemotherapy delays are important prognostic factors for survival and these modifications should be minimized.

## Conclusions

Anthracycline dose reduction in patients with stage I- III breast cancer were associated with higher mortality risk and significantly decreased 5- year absolute survival in all molecular subtypes. Chemotherapy delays alone were not associated with decreased survival only in HER2 positive subtype. The most common reason for dose reduction or chemotherapy delays was neutropenia.

## Additional file

Additional file 1: Table S1. Cox proportional hazards model on 5-year survival when impact of tumor size, molecular subtype and menopausal status were adjusted in four patients groups. (DOC 31 kb)

## Abbreviations

AC: doxorubicin, cyclophosphamide
CI: confidence interval
CMF: cyclophosphamide, methotrexate, fluorouracil
ER: estrogen receptor
FAC: fluorouracil, doxorubicin, cyclophosphamide
HER: human epidermal receptor
HR: hazard ratio
OS: overall survival
RDI: relative dose intensity
RR: relative risk
