# Supplementary material for: Retrospective analysis of the impact of anthracycline dose reduction and chemotherapy delays on the outcomes of early breast cancer molecular subtypes
Source: BMC Cancer. 2018 Apr 20;18:453. doi: 10.1186/s12885-018-4365-y (PMC5910571; doi:10.1186/s12885-018-4365-y)
Supplement: Supplementary file 1 — Table S1. Cox proportional hazards model on 5-year survival when impact of tumor size, molecular subtype and menopausal status were adjusted in four patients groups. (DOC 31 kb) [file 12885_2018_4365_MOESM1_ESM.doc]

**Additional file 1:** Cox proportional hazards model.

**Additional file 1: Table S1.** Cox proportional hazards model on 5-year survival when impact of tumor size, molecular subtype and menopausal status were adjusted in four patients groups.

|  | | | | | | | | |
| --- | --- | --- | --- | --- | --- | --- | --- | --- |
|  | B | SE | Wald | df | Sig. | Exp(B) | 95,0% CI for Exp(B) | |
|  |  |  |  |  |  |  | Lower | Upper |
| No reductions and delays |  |  | 15,609 | 3 | ,001 |  |  |  |
| Reductions only | 1,154 | ,324 | 12,668 | 1 | ,000 | 3,171 | 1,679 | 5,985 |
| Delays only | ,345 | ,395 | ,762 | 1 | ,383 | 1,412 | ,651 | 3,061 |
| Both delays and reductions | 1,015 | ,366 | 7,685 | 1 | ,006 | 2,761 | 1,346 | 5,660 |
